# Thalidomide Suppresses Angiogenesis Through the Signal Transducer and Activator of Transcription 3/SP4 Signaling Pathway in the Peritoneal Membrane

**DOI:** 10.3389/fphys.2021.712147

**Published:** 2021-09-03

**Authors:** Nan Zhu, Ling Wang, Huimin Guo, Jieshuang Jia, Lijie Gu, Xuan Wang, Man Yang, Haochen Guan, Weijie Yuan

**Affiliations:** ^1^Department of Nephrology, Shanghai General Hospital, Shanghai, China; ^2^Department of Nuclear Medicine, Shandong Provincial Hospital Affiliated to Shandong First Medical University, Shandong, China

**Keywords:** peritoneal dialysis, angiogenesis, thalidomide, STAT3, SP4

## Abstract

Peritoneal angiogenesis is the key pathophysiological factor that limits peritoneal ultrafiltration during peritoneal dialysis (PD) in uremic patients. Thalidomide has been confirmed to inhibit angiogenesis by inhibiting the secretion of vascular endothelial growth factor (VEGF), but the exact mechanism by which thalidomide inhibits vascular proliferation during PD is still unclear. Here, the objective of the present study was to investigate whether the reduction in VEGF production by human peritoneal mesothelial cells (HPMCs) was controlled by thalidomide. Stimulation of HPMCs with IL-6 in combination with soluble IL-6 receptor (sIL-6R) promoted VEGF expression and secretion, but these effects were attenuated by thalidomide treatment through a transcriptional mechanism that involved signal transducer and activator of transcription 3 (STAT3) and SP4. Conditioned medium from HPMCs cultured with thalidomide inhibited angiogenic endothelial tube formation, which could be further blocked by silencing SP4 and promoted by overexpressing SP4. *In vivo*, induction of peritoneal angiogenesis in sham rats, sham+PD rats, 5/6 nephrectomy (5/6Nx) rats, 5/6Nx+PD rats, and 5/6Nx+PD rats intraperitoneally treated with thalidomide showed that thalidomide was involved in the control of several key endothelial–specific targets, including *VEGFR2*, *VEGFR3*, *SP4*, and *STAT3* expression and new vessel formation, confirming the role of thalidomide and STAT3/SP4 signaling in these processes. Taken together, these findings identify a novel mechanism that links thalidomide, STAT3/SP4 signaling, and angiogenesis in the peritoneal membrane.

## Introduction

Peritoneal dialysis (PD) is an important component of replacement therapy for end-stage renal disease (ESRD), and PD provides the possibility of long-term survival for patients with uremia. However, over time, the withdrawal rate of patients gradually increases, mainly due to ultrafiltration failure (UFF; Smit et al., 2008). The incidence of peritoneal UFF is as high as 36%, especially in patients with a penetration age of more than 6years (Taki et al., 2017). Therefore, peritoneal UFF is a serious problem encountered by long-term peritoneal dialysis patients.

Angiogenesis is a structural change in the peritoneum that has attracted substantial attention in recent years (Capobianco et al., 2017). Pathological studies of human peritoneal biopsies suggested that the peritoneal vascularization density of patients with peritoneal failure was significantly higher than that of control subjects (Sherif et al., 2006). Studies of animal models have also confirmed that peritoneal vascular proliferation is obvious during peritoneal dialysis in uremic patients and that the degree of peritoneal vascularization is negatively correlated with the amount of ultrafiltration. After inhibiting angiogenesis, the amount of peritoneal ultrafiltration significantly increases (Stavenuiter et al., 2011). Peritoneal angiogenesis plays a dominant role in the pathogenesis of peritoneal UFF. The specific mechanism is that peritoneal angiogenesis leads to increased surface area of peritoneal blood vessels, increased area coefficient of peritoneal material transport, and increased and faster absorption of glucose; these effects accelerate the transport of small molecular solutes and cause the rapid disappearance of peritoneal osmotic gradient, resulting in loss of peritoneal ultrafiltration function and decreasing ultrafiltration (Rippe, 2009). Therefore, an in-depth understanding of the occurrence and development of peritoneal neovascularization during dialysis is of great significance for the prevention and treatment of peritoneal failure and the improvement of the survival rate and quality of life of patients undergoing long-term peritoneal dialysis.

Vascular endothelial growth factor (VEGF) is currently recognized as the main factor that mediates angiogenesis. VEGF can increase the permeability of blood vessels and promote the proliferation of vascular endothelial cells and angiogenesis (Apte et al., 2019). Scholars have conducted pathological biopsy studies on the peritoneal tissue of peritoneal dialysis patients, and the results show that the number of peritoneal interstitial blood vessels in peritoneal dialysis patients is positively correlated with the amount of VEGF in peritoneal dialysate (Karaman et al., 2018). The results of animal experiments also confirmed that there are large numbers of new capillaries in the peritoneal tissue of rats after long-term dialysis. Intraperitoneal injection of an anti-VEGF neutralizing antibody can significantly suppress the formation of capillaries in rat peritoneal tissue and the peritoneal interstitium as well as the degree of thickening (Io et al., 2004). Based on this finding, VEGF was directly demonstrated to play an important role in peritoneal angiogenesis.

Signal transducer and activator of transcription 3 (STAT3) is the Janus kinase (JAK) substrate of the JAK-STAT3 pathway. STAT3 participates in many important biological processes in the human body, such as differentiation, proliferation, angiogenesis, and cellular apoptosis, and plays a very important role in the signal transduction of cytokines (Guanizo et al., 2018). Studies have shown that after IL-6 and gp130 form a dimer, this dimer can activate JAK, which is associated with gp130, thereby activating the receptor tyrosine kinase. The phosphorylated residue binds to, phosphorylates, and activates the STAT3 protein. The activated STAT3 monomer forms a dimer, translocates to the nucleus, modifies the expression of target genes by binding to the target gene, and participates in cell proliferation, survival, transformation, migration, and other processes (Galoczova et al., 2018). Studies have shown that IL-6/soluble IL-6 receptor (sIL-6R) can activate STAT3 to induce VEGF expression, and the trend is similar to that of SP4. Silencing STAT3 and SP4 decreases VEGF expression (Catar et al., 2017). Therefore, the STAT3/SP4 transcription axis may play an important regulatory role in the process of IL-6 complex-induced VEGF expression.

Thalidomide is clinically used as a drug to treat multiple myeloma and is known to have anti-angiogenic effects. Arai et al. (2011) injected chlorhexidine gluconate (CG) into the abdominal cavity of mice, orally administered thalidomide, and observed that the peritoneum without thalidomide showed thickening of the submesothelial area, blood vessels, and muscles. The number of fibroblasts increased (Kenyon et al., 1997; Arai et al., 2011). Many VEGF-, proliferating cell nuclear antigen (PCNA)-, and TGF-β-positive cells were observed in the submesothelial area. Thalidomide treatment significantly decreased submesothelial thickening and angiogenesis and reduced the number of cells expressing PCNA and VEGF, myofibroblasts, and TGF-β-positive cells (Zhang et al., 2008; Arai et al., 2011). In addition, thalidomide weakened the permeability of the peritoneum to creatinine compared with the control. Therefore, thalidomide has potential anti-peritoneal angiogenesis effects.

Based on the results of previous studies and the discussion above, we speculate that thaildomide may inhibit VEGF induced peritoneal angiogenesis *via* transcription factors STAT3/SP4. Inhibiting peritoneal angiogenesis could delay peritoneal dialysis membrane failure and prolong time on peritoneal dialysis. Thus, our study could provide new treatment options for patients with membrane failure and expand the range of therapeutic applications of thalidomide.

## Materials and Methods

### Cell Culture and Treatment

Human peritoneal mesothelial cells (HPMCs) were purchased from the American Type Culture Collection (ATCC) and cultivated in Dulbecco’s modified Eagle’s medium (DMEM) with 10% fetal bovine serum (FBS; Thermo Fisher Scientific, Waltham, MA, United States), 100U/ml penicillin, and 100mg/ml streptomycin (Thermo Fisher Scientific). The cultures were incubated at 37°C in a humidified CO_2_ (5%) atmosphere. The HPMCs were treated with 100ng/ml IL-6+sIL-6R for 24h and 1, 10, and 100μM thalidomide for 6, 12, or 24h prior to the subsequent assays. The final volume was 200μl, and the thalidomide control group was treated with DMSO alone.

### Plasmid Construction and Cell Transfection

Human peritoneal mesothelial cells were transiently transfected with STAT3 siRNAs and SP4 siRNA after being cultured in six-well plates overnight. A mixed negative control, namely, an empty vector, was transfected by applying the Lipofectamine 2000 transfection reagent (Thermo Fisher Scientific) and FuGENE® HD Transfection Reagent (Roche, Wetzlar, Germany), following the manufacturer’s instructions. The cells were then harvested to observe the knockout efficiency *via* quantitative real-time PCR (qRT-PCR) 48h after transfection. Three distinct siRNAs against STAT3 or SP4 were designed and synthesized by GenePharma (Shanghai, China). The siRNA sequence against STAT3 was 5'-GCTCAACGAGTGCTTCATCAAGCTACCCA-3'. The siRNA sequence against SP4 was 5'-AAGTTGTAGTTGTTTGGAAATAT-3'.

### Quantitative Real-Time PCR

Total RNA was extracted from cells and tissues with TRIzol reagent as stated by the protocol. All the mRNA was subjected to quantitative polymerase reaction and reverse transcription according to the protocol for the PrimeScript® RT Master Mix Perfect Real Time (TAKARA Bio Inc., Kusatsu, Japan) and SYBR Green Master Mix (Applied Biosystems, Foster City, CA, United States), respectively. The qPCR reaction was conducted on an Applied Biosystems 7900HT Real-Time System. The primer sequence information was showed in Supplementary Table 1.

### Western Blot Analysis

The collected cells were lysed in RIPA protein extraction reagent (Beyotime, Beijing, China) supplied with a cocktail of protease inhibitors (Roche, Pleasanton, CA, United States). The lysis products were loaded onto sodium dodecyl sulfate (SDS)-polyacrylamide gel electrophoresis (PAGE) gels for electrophoresis, transferred to polyvinylidene fluoride (PVDF) membranes, and blocked in 5% milk prior to incubation with the indicated primary antibodies and secondary antibodies. Autoradiograms were quantified by densitometry, and glyceraldehyde 3-phosphate dehydrogenase (GAPDH) was used as a control. Antibodies against VEGFR2, SP4, and STAT3 were purchased from Abcam (Cambridge, United Kingdom).

### Enzyme Linked Immunosorbent Assay

The intracellular VEGF release was detected using the enzyme linked immunosorbent assay (ELISA) kit (Solarbo, Beijing) according to the manufacturer’s instructions.

### Endothelial Cell Culture and Tube Formation Assay

Human umbilical vein endothelial cells (HUVECs) were purchased from ATCC. For the tube formation assay, Matrigel (BD Biosciences, San Diego, CA, United States) was added to a 96-well plate (50μl per well) and solidified at 37°C for 30min. HUVECs (2×10^4^ cells per well) were seeded onto the Matrigel and cultured in DMEM with or without 10% (vol/vol) conditioned medium from HPMCs treated as described in the figures. The total length of the tube-like structures was measured 24h later under a microscope (Olympus, Japan), and five randomly selected fields from each well were analyzed to determine the total capillary length using ImageJ 1.43 software.56.

### Animal Experiments

All the animal procedures were performed under appropriate licenses and according to institutional animal care guidelines. The rats were purchased from Vital River Laboratory Animal Technology (Beijing, China). All animals were housed in individual ventilated cages, provided sterilized water and food at libitum, and handled under specific pathogen-free conditions in the Institute’s animal care facilities, which meet international standards. Rats underwent 5/6 Nx or sham surgery under ketamine/xylazine anesthesia (100mg/kg). Briefly, the left kidney was exposed, and the upper and lower poles were tied with a polyglycolic acid suture line, followed by right nephrectomy. The peritoneum and skin were then sutured, and the animals were returned to their individual cages. One week later, the sham and 5/6 Nx rats were injected with 20ml of peritoneal dialysate (3.86% glucose) daily for 4weeks. And then the 5/6 Nx+PD rats were injected with thalidomide (200mg/kg/day). The control group was intraperitoneally injected with normal saline. The rats were euthanized at the designated time points (12, 18, and 24, respectively), and specimens of peritoneal peritoneum were collected and snap frozen in liquid nitrogen.

### Immunohistochemistry

Paraffin-embedded blocks were cut into 4-μm-thick sections and dewaxed and hydrated. Then, the sections were immersed in distilled water containing 3% hydrogen peroxidase twice to reduce the endogenous oxidase activity. Next, the tissue sections were incubated with CD34 antibodies (Abcam, ab81289) for 2h at room temperature, and subsequently, a goat-anti-rabbit antibody was applied to the cells at room temperature for 40min. The degree of staining was developed by diaminobenzidine (DAB) chromogen (BioRad, Inc., CA, United States). Subsequently, the tissues were dehydrated and sealed with gum. Five random fields of view were selected at 100× magnification with a camera using a microscope (Olympus, Japan), and the mean of the microvessel counts was recorded as the microvessel density.

### Statistical Analysis

All the statistical analyses were executed using SPSS 22.0 software (IBM Corporation, Armonk, NY, United States) and GraphPad Prism 5.0 (GraphPad Inc., La Jolla, CA, United States). Differences between groups were analyzed utilizing Student’s *t*-test or one-way ANOVA. Values of *p*<0.05 were considered to be statistically significant.

## Results

### Thalidomide Treatment Modulates the Expression of SP4, STAT3, and VEGFR2 After Treatment of HPMCs With IL-6 and sIL-6R

**Figure 1:**
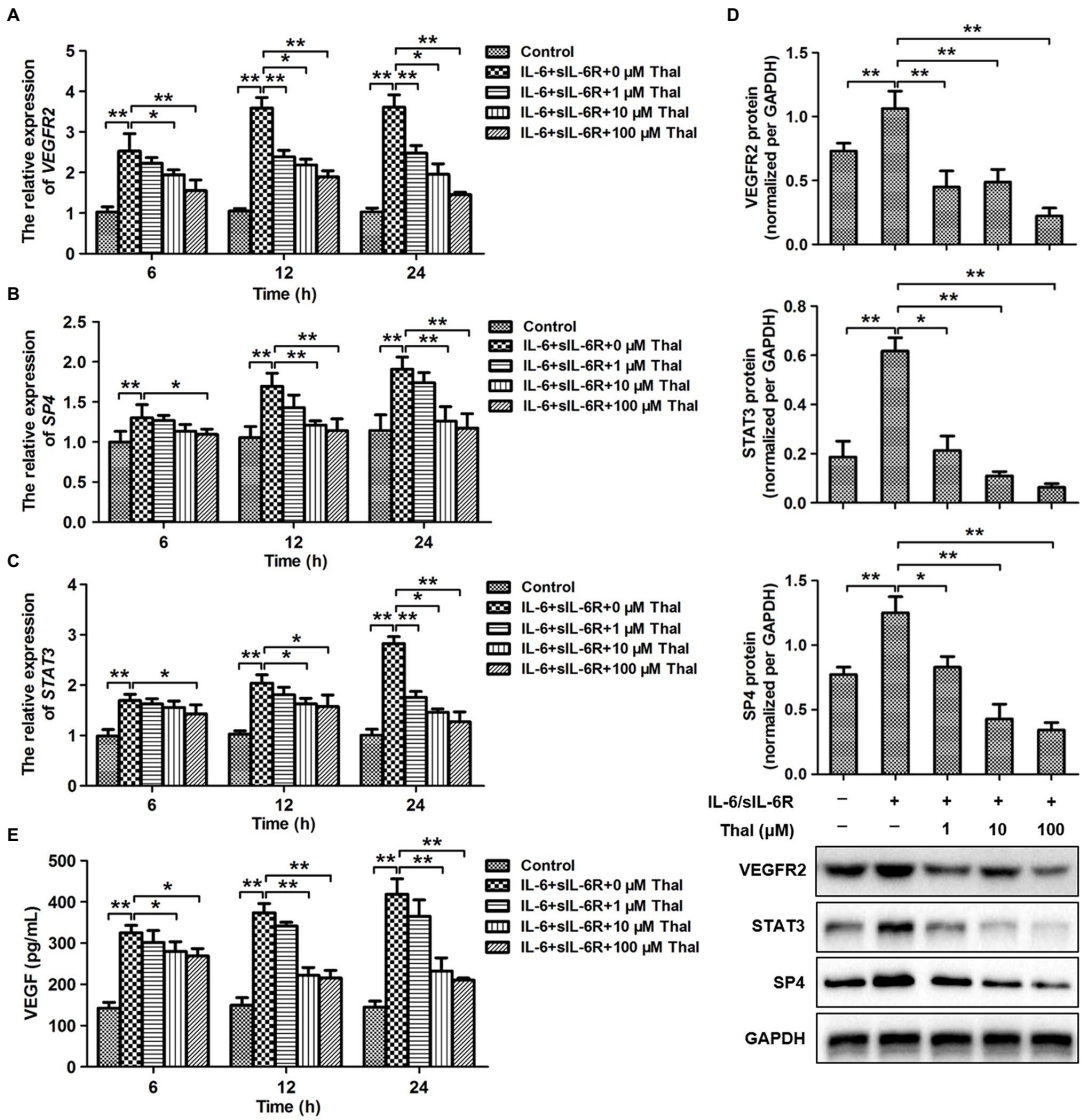
Effect of thalidomide on the expression of VEGFR2, SP4, and signal transducer and activator of transcription 3 (STAT3) in human peritoneal mesothelial cells (HPMCs) treated with a combination of IL-6 and soluble IL-6 receptor (sIL-6R). **(A–C)** quantitative real-time PCR (qRT-PCR) analyses were performed to evaluate the expression of VEGFR2, SP4, and STAT3 in HPMCs treated with IL-6+sIL-6R (both at 100ng/ml) at three time points (6, 12, and 24h) in the presence of thalidomide at dose concentrations of 0, 1, 10, and 100μM. **(D)** The western blot results showed that the protein levels of VEGFR2, SP4, and STAT3 changed in HPMCs treated with IL-6+sIL-6R (both at 100ng/ml) at 48h in the presence of thalidomide at dose concentrations of 0, 1, 10, and 100μM. For the quantitative analysis of VEGFR2, SP4, and STAT3 protein levels, the target protein expression was normalized to that of glyceraldehyde 3-phosphate dehydrogenase (GAPDH). **(E)** The intracellular content of vascular endothelial growth factor (VEGF) from enzyme linked immunosorbent assay (ELISA). ^*^p<0.05; ^**^p<0.01. Thal, Thalidomide.

According to previous results, the stimulation of HPMCs with IL-6 in combination with sIL-6R promoted VEGFR2 expression through a transcriptional mechanism involving STAT3 and SP4. To test whether treatment with thalidomide may modulate VEGF induction by regulating the expression of the transcription factor STAT3 and SP4, we analyzed the peritoneum mRNA levels of SP4, STAT3, and VEGFR2. Indeed, simultaneous exposure to IL-6 plus sIL-6R with 6, 12, and 24h resulted in significant increases of SP4, STAT3, and VEGFR2 expression compared to the control group (^**^p<0.01). In addition, thalidomide treatment may attenuate the mRNA levels of SP4, STAT3, and VEGFR2 in a significant time- and dose-dependent manner (Figures 1A–C). The greatest effect was achieved with thalidomide at a dose of 100μM for 24h, but 10μM thalidomide treatment for 12 and 24h also revealed significant reducing of SP4, STAT3, and VEGFR2 expressions (^*^p<0.05; ^**^p<0.01). Furthermore, western blot analysis showed that SP4, STAT3, and VEGFR2 protein levels increased significantly under IL-6 plus sIL-6R treatment and decreased obviously after thalidomide supplementation in the IL-6 plus sIL-6R solution for 48h (^**^p<0.01; Figure 1D). The intracellular content of VEGF from ELISA analysis was increased significantly with the IL-6 plus sIL-6R treatment, and decreased similarly to the SP4, STAT3, and VEGFR2 expressions with thalidomide supplementation (^**^p<0.01; Figure 1E). These observations prompted us to examine the signaling events underlying thalidomide treatment in more detail.

### Effect of Thalidomide on STAT3/SP4 Blockade or Overexpression in HPMCs

**Figure 2:**
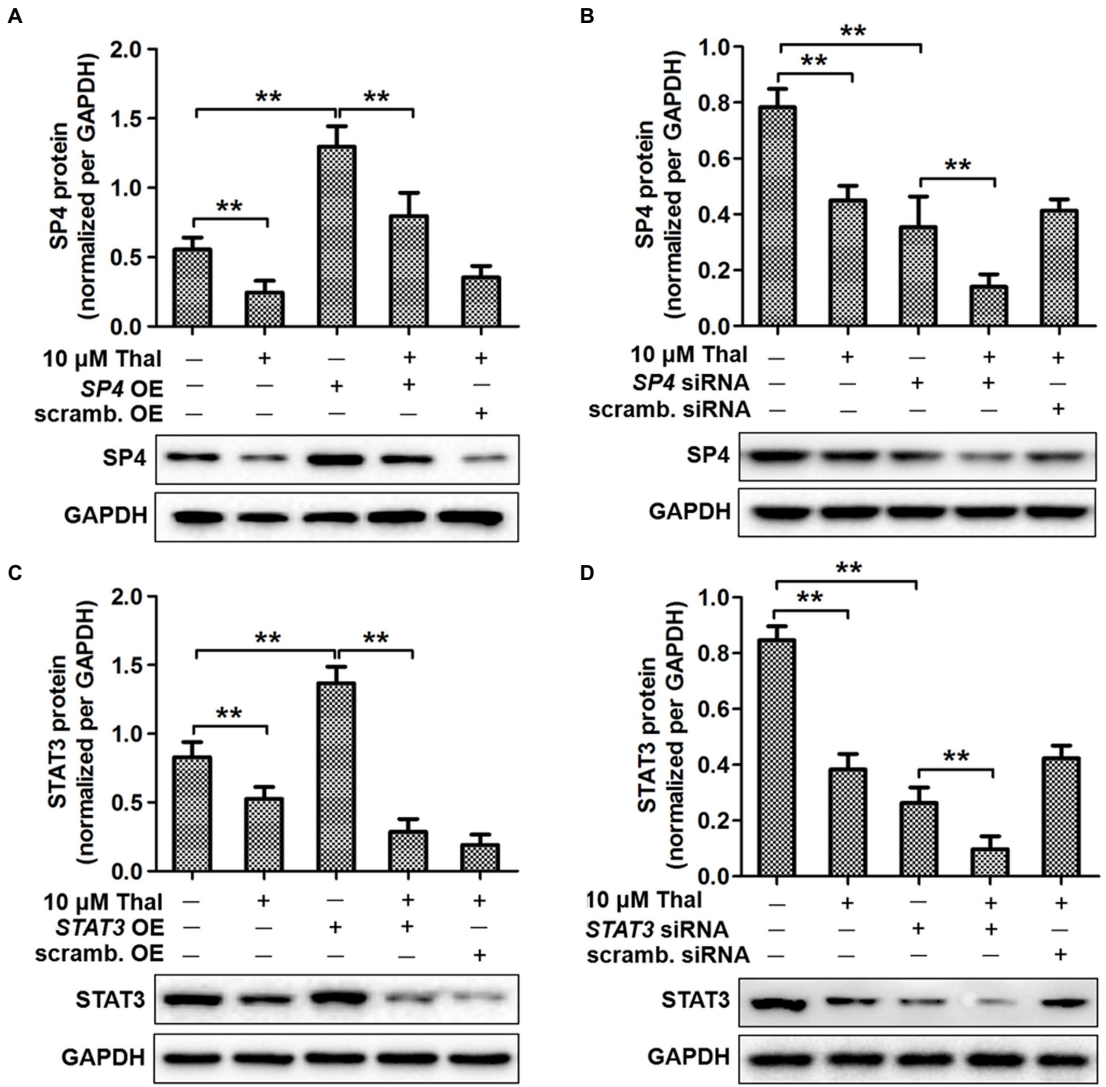
The role of STAT3 and SP4 in SP4–mediated VEGF induction by thalidomide. Effects of SP4 overexpression **(A)** and silencing **(B)** on SP4 expression. Cells were transiently transfected with either SP4 OE or scrambled (scrambled.) OE and SP4 siRNA or scrambled (scrambled.) siRNA. Then, the cells were stimulated with thalidomide (10μM) for 24h. The expression of STAT3 was analyzed in cells transfected with either STAT3 OE **(C)** or STAT3 siRNA **(D)**. Cells were assessed for SP4 and STAT3 protein expression by Western blotting. Quantitative analysis of SP4 and STAT3 protein expression was normalized to GAPDH expression. ^*^p<0.05; ^**^p<0.01.

To further determine whether there is a link between thalidomide and STAT3/SP4, the expression of the STAT3 and SP4 was blocked by RNA interference or overexpressed in HPMCs. These results showed that the protein levels of SP4 and STAT3 were significantly decreased in response to thalidomide treatment compared with control treatment (^**^p<0.01; Figures 2A,D). The protein levels of SP4 and STAT3 increased, respectively under their respective overexpression conditions (^**^p<0.01; Figures 2A,C). But thalidomide significantly suppressed their protein expressions compared to the SP4 or STAT3 overexpression conditions (^**^p<0.01; Figures 2A,C). In addition, SP4- or STAT3-targeting siRNA, but not a scrambled siRNA control, inhibited SP4 or STAT3 expressions, respectively at the protein level (^**^p<0.01; Figures 2B,D). The thalidomide supplement further inhibited SP4 or STAT3 expression under SP4 or STAT3-targeting siRNA conditions (^**^p<0.01; Figures 2B,D).

### Effect of Thalidomide on VEGF Production and Angiogenesis

**Figure 3:**
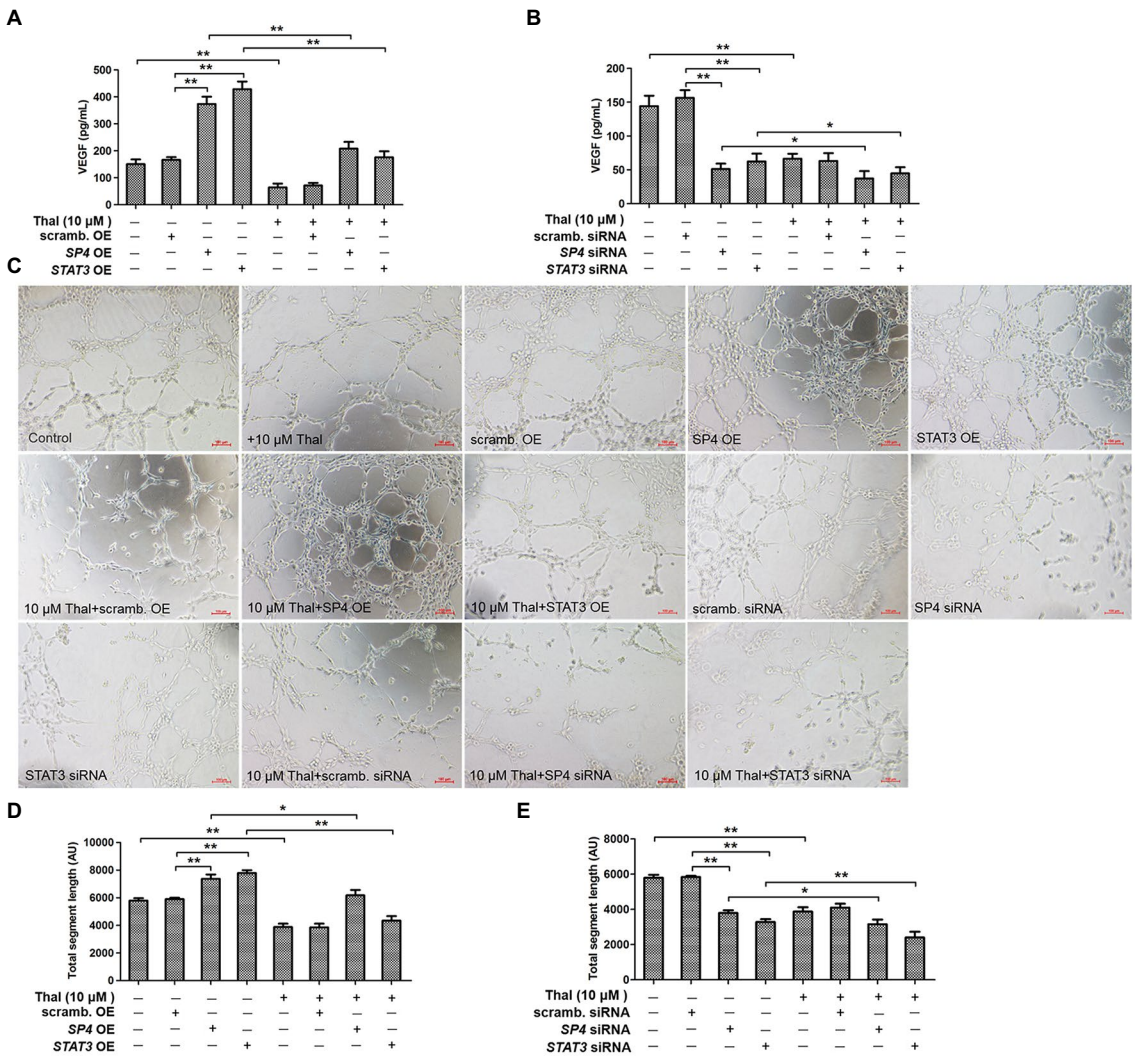
Effect of thalidomide on VEGF–mediated endothelial cell tube formation. VEGF secretion was assessed in HPMCs transiently transfected with either SP4-OE, STAT3-OE, or scramb. OE **(A)**, either SP4 siRNA, STAT3 siRNA, or scramb. siRNA **(B)**, stimulated with thalidomide (10μM) for 24h. **(C)** Effect of conditioned medium (10% vol/vol) from HPMCs treated as in **(A,B)** on endothelial cell tube formation within 16h. **(D,E)** Quantification of total segment length is presented in order corresponding to experimental groups as shown in **(C)**. ^*^p<0.05; ^**^p<0.01.

To further confirm the involvement of thalidomide in VEGF production and biological activity, HPMCs were stimulated with thalidomide in the presence of either SP4 or STAT3 overexpression or interference (Figure 3). VEGF productions were significantly decreased with thalidomide treatment (^**^p<0.01), but significantly increased in SP4 or STAT3 overexpressing group and decreased in SP4 or STAT3 siRNA group when compared to the scrambled controls (^**^p<0.01; Figures 3A,B). Moreover, VEGF production in SP4 OE+Thal group was significantly decreased when compared to the SP4 OE group (^**^p<0.01; Figure 3A). On the contrary, SP4 siRNA plus thalidomide treatment resulted in a significant inhibition of VEGF protein release, compared to the SP4 siRNA group (^*^p<0.05; Figure 3B). The thalidomide treatment under STAT3 OE or STAT3 siRNA conditions revealed the similar effects on VEGF release (^*^p<0.05; ^**^p<0.01; Figures 3A,B). To test the functional properties of VEGF production, conditioned medium from stimulated HPMCs was transferred to endothelial cell cultures, and the formation of capillaries was assessed. Incubation of HUVECs in the presence of conditioned medium from thalidomide–stimulated HPMCs significantly decreased endothelial cell tube formation (^**^p<0.01; Figures 3C–E). A similar degree of stimulations were observed when conditioned medium from HPMCs treated with scrambled OE+Thal or scrambled siRNA+Thal were used (Figures 3C,D). The endothelial cell angiogenesis was significantly increased in response to conditioned medium from HPMCs with SP4 or STAT3 overexpressing compared to the scrambled OE group (^**^p<0.01), but significantly decreased in conditioned medium from SP4 OE+Thal or STAT3 OE+Thal groups compared to the corresponding OE groups (^*^p<0.05; ^**^p<0.01; Figures 3C,D). In addition, the endothelial cell angiogenesis was significantly decreased in conditioned medium from SP4 or STAT3 siRNA compared to the scrambled siRNA group (^**^p<0.01), and further decreased in the medium with SP4 siRNA+Thal or STAT3 siRNA+Thal (^*^p<0.05; Figures 3C,E).

### Effect of Thalidomide on Peritoneal Expression of Genes Essential for VEGF Activity in Rats

**Figure 4:**
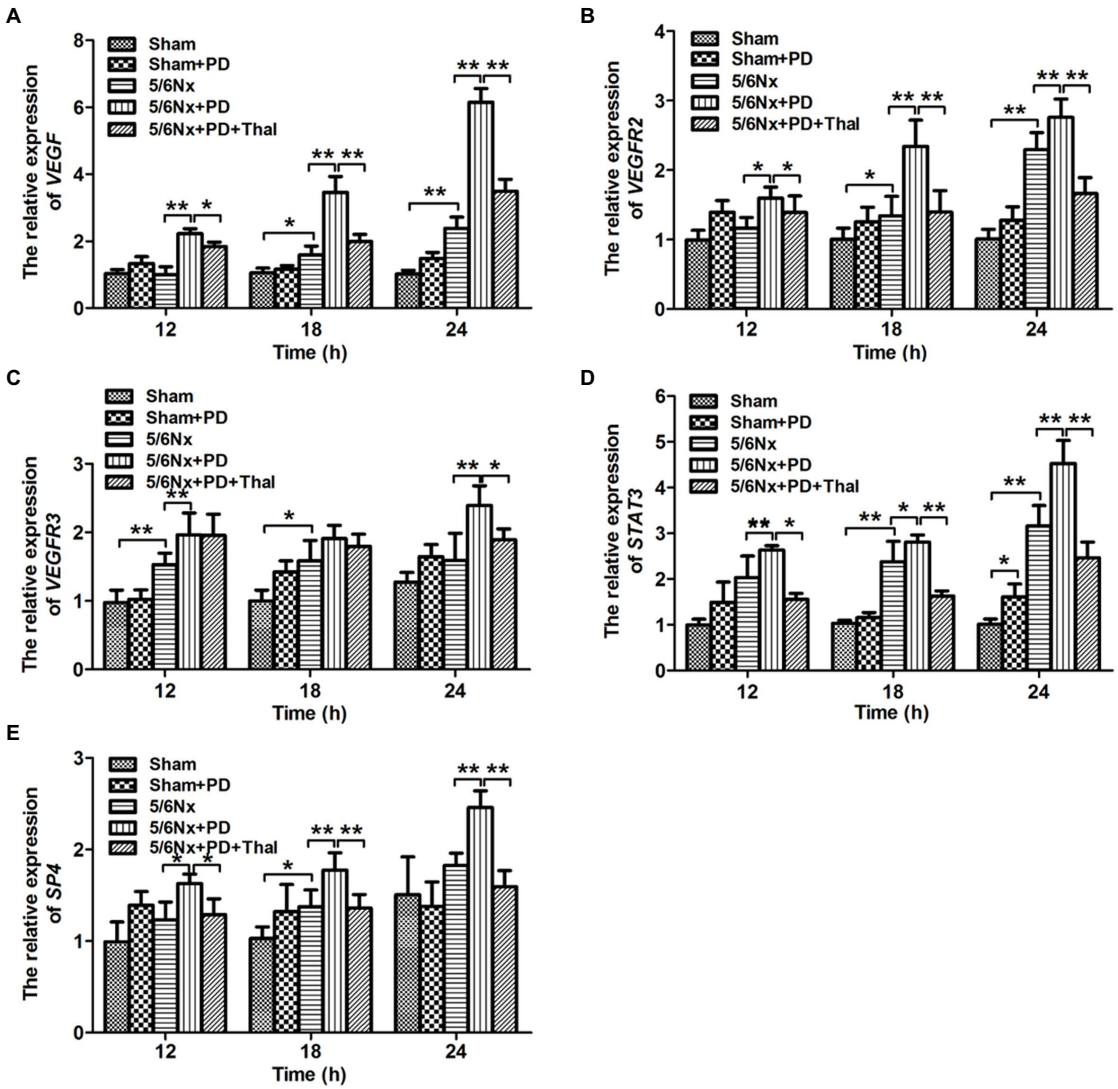
Effect of thalidomide on peritoneal expression of endothelial-specific targets in 5/6Nx+PD model rats. qRT-PCR analyses were performed to evaluate the peritoneal expression of *VEGF* **(A)**, *VEGFR2* **(B)**, *VEGFR3* **(C)**, *STAT3* **(D)**, and *SP4* **(E)** in sham, sham+PD, 5/6Nx, 5/6Nx+PD, and 5/6Nx+PD rats intraperitoneally administered thalidomide at the indicated time points (12, 18, and 24h). ^*^p<0.05; ^**^p<0.01.

To determine whether decreased peritoneal VEGF due to thalidomide can initiate events leading to inhibited vascular permeability and angiogenesis, we examined the mRNA expression of several key endothelial–specific targets involved in these processes in sham, sham+PD, 5/6Nx, 5/6Nx+PD, and 5/6Nx+PD+Thal rats at defined time points (12, 18, and 24h). These targets included *VEGF*, *VEGFR2*, *VEGFR3*, *STAT3*, and *SP4*. The expression of all these angiogenesis-related targets was significantly increased within 8h in the 5/6Nx+PD rats, compared to the sham+PD rats (^**^p<0.01; Figures 4A–E). To assess whether this effect was related to thalidomide activity, the 5/6Nx+PD rats were treated with thalidomide. The addition of thalidomide significantly decreased *VEGF*, *VEGFR2*, *VEGFR3*, *STAT3*, and *SP4* expressions in the inflamed peritoneum of 5/6Nx+PD rats (Figures 4A–E).

### Effect of Thalidomide on Microvessel Density in Rats

**Figure 5:**
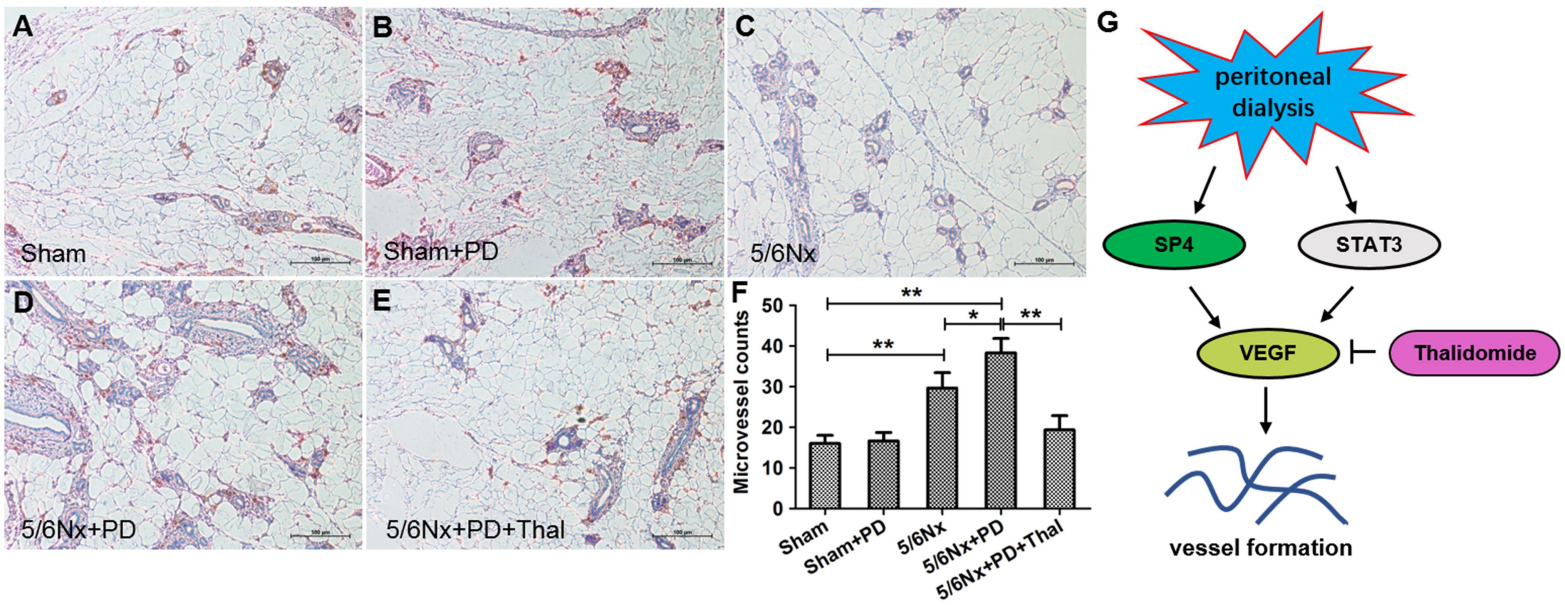
Thalidomide reduced microvessel density in 5/6Nx+PD model rats. Microvessel density (marker: CD34) was determined by IHC assay in the peritoneum of rats. **(A)** sham rats, **(B)** sham+PD, **(C)** 5/6Nx rats, **(D)** 5/6Nx+PD rats, and **(E)** 5/6Nx+PD rats were intraperitoneally administered thalidomide. **(F)** Quantitative analysis of the microvessel counts to calculate the microvascular density. **(G)** Proposed scheme for the mechanism by which thalidomide obstructs vessel formation *in vivo* and *in vitro*. Each of the four groups contained seven rats (*n*=7). Bars=100μm. ^*^p < 0.05; ^**^p < 0.01.

To investigate the relationship between microvessel density and thalidomide treatment in the peritoneum, immunohistochemistry was performed to assess the positive CD34 signalling in the sham, sham+PD, 5/6Nx, 5/6Nx+PD, and 5/6Nx+PD+Thal rats (Figures 5A–E). CD34-positive cells were mainly observed in the 5/6Nx rats and 5/6Nx+PD rats (Figures 5C,D). The microvascular density quantified by microvessel counts was significantly increased in the peritoneum of the 5/6Nx+PD rats and decreased in the 5/6Nx+PD rats treated with thalidomide (^*^p<0.05; ^**^p<0.01; Figure 5F).

## Discussion

Over time, the incidence of ultrafiltraiton failure on peritoneal dialysis gradually increases. The peritoeneum is subject to ongoing inflammation (Bastug et al., 2013). Therefore, delaying the occurrence of peritoneal UFF is particularly important. Peritoneal angiogenesis is the key pathophysiological basis of peritoneal UFF during peritoneal dialysis in uremic patients. The formation of new blood vessels increases the exchange area between blood and peritoneal dialysate, increases the transport of small molecules through the peritoneum, and ultimately damages the ultrafiltration function of the peritoneum (Twardowski, 2006). VEGF was originally isolated from bovine pituitary acinar cell culture and specifically stimulated the division of vascular endothelial cells and promote angiogenesis. In the process of peritoneal UFF, mesothelial cells and capillary endothelial cells can secrete VEGF, leading to neovascularization and increased peritoneal capillary permeability (Kim, 2009). IL-6 is a multipotent cytokine. It was reported that the increase of the IL-6 content in the peritoneal fluid of long-term peritoneal dialysis patients was correlated with the expression of peritoneal solute transport rates (PSTR) and VEGF (Yang et al., 2017; Guanizo et al., 2018). Both IL-6 and sIL-6R can be detected in the dialysate of long-term peritoneal dialysis patients (Le Meur et al., 1999). Our research found that *in vitro*, the use of IL-6 plus sIL-6R significantly stimulated HPMCs to secrete VEGF. This result is consistent with the results of reported *in vitro* studies (Hashizume et al., 2009).

Thalidomide is a synthetic glutamate derivative. At present, many studies have found that thalidomide plays an important role in immune regulation, anti-inflammatory activity, anti-angiogenesis activity, and collagen synthesis inhibition. Scholar Verheul et al. (1999) reported that thalidomide has anti-angiogenic properties and can inhibit VEGF expression. Andersen et al. (2012) found that VEGF gene polymorphisms in patients with multiple myeloma are related to the therapeutic effects of the antiangiogenic drug thalidomide. Thalidomide is relatively ineffective in multiple myeloma patients with the ACG haplotype in rs699947, rs833061, and rs2010963 loci. Bauditz reported that thalidomide can be used to treat myeloma and Crohn’s disease because it can inhibit inflammation and regulate immune responses (Bauditz, 2016). Thalidomide is a drug that can inhibit angiogenesis. Studies have reported that thalidomide treatment is used to evaluate the effects of VEGF expression and angiogenesis in psoriasis, and it is concluded that thalidomide can improve psoriasis-like lesions in an imivomod-induced psoriasis model in a dose-dependent manner and inhibit the expression of VEGF in the skin (Liu et al., 2018). In this study, we used IL-6 plus sIL-6R to activate HPMCs. We added different concentrations of thalidomide to the treatment and found that as the thalidomide concentration increased, the expression of VEGFR2 significantly decreased, indicating that thalidomide can significantly inhibit the expression of VEGFR2. In addition, the expressions of SP4 and STAT3 were also reduced in a time and dose-dependent manner with thalidomide supplement. It was found that the transcription factor SP4 can target VEGF. And according to the results from ELISA, the intracellular content of VEGF also decreased in a dose-dependent manner with thalidomide treatment. Therefore, we speculate that thalidomide may regulate the expression of VEGF through the STAT3/SP4 signaling pathway. Some scholars have also discovered *in vitro* that thalidomide can bind to fibroblast growth factor receptor 3 (FGFR3) to activate extracellular signal regulated kinases (ERK) and phosphorylate STAT3. The signal transduction pathway causes the expression of VEGF to be downregulated, and this inhibitory effect is obvious with the increase in the concentration of thalidomide, showing a dose-dependent change (Qian et al., 2005). Our results show that thalidomide inhibits VEGF and decreases STAT3/SP4. In the STAT3 or SP4 overexpressing groups, thalidomide addition significantly reduced the cytosolic VEGF content. And thalidomide showed further inhibition effect in STAT3 or SP4 siRNA groups. Thus, thalidomide appeared upstream of VEGF, and exerted antagonistic effects *via* STAT3/SP4 signaling.

Studies on human endothelial cells have also found that thalidomide can not only reduce the level of VEGF production but also inhibit VEGF-induced cell migration, capillary formation, and angiogenesis (Komorowski et al., 2006). The formation process of peritoneal neovascularization is very similar to the formation of neovascularization in tumor tissues. Both of the processes involve proliferation and migration to form blood vessels through the differentiation and maturation of vascular endothelial cells in existing blood vessels. That is, angiogenesis is the main mechanism of peritoneal neovascularization. Our research found that after enhancing the SP4 signaling pathway, HPMCs can secrete VEGF and promote blood vessel formation. The addition of thalidomide can significantly reduce the secretion of VEGF and inhibit the formation of blood vessels. The results show that thalidomide suppresses the formation of peritoneal neovascularization through inhibiting the STAT3 or SP4 signaling pathway (Figure 5G). In this study, we established a nephrectomy uremic rat (Wistar) peritoneal dialysis animal model and injected thalidomide into the abdominal cavity to observe the effect of thalidomide on inhibiting neovascularization *in vivo*. Our results show that thalidomide can significantly reduce the expression of VEGF, VEGFR2, VEGFR3, STAT3, and SP4 in the peritoneum of nephrectomy uremic rats after peritoneal dialysis and significantly inhibit peritoneal neovascularization. The decreased microvessel density in the thalidomide-treated 5/6Nx+PD rats suggested that suppressive angiogenesis might account for the long presence of peritoneum observed in the process of peritoneal dialysate following thalidomide administration. The results described above further indicate that thalidomide can be used as a drug to inhibit angiogenesis, inhibit the expression of VEGF in peritoneal tissue, and delay the progression of peritoneal neovascularization.

Taken together, these results demonstrate that thalidomide treatment reduced the expression of *VEGFR2*, *STAT3*, and *SP4* in HPMCs treated with IL-6 combined with sIL-6R. Moreover, angiogenic endothelial tube formation was inhibited by conditioned medium from HPMCs cultured with thalidomide, which could be further blocked by silencing SP4 or STAT3 and promoted by overexpressing SP4 or STAT3. *In vivo*, qRT-PCR analysis revealed that thalidomide attenuated the expression of several key endothelial-specific targets; including *VEGFR2*, *VEGFR3*, *SP4*, and *STAT3*, and immunohistochemistry showed that thalidomide inhibited the formation of new vessels in 5/6Nx+PD rats. Finally, these observations may help clarify the possible therapeutic actions of thalidomide in uremic patients with PD. In addition, SP1 as a transcription factor binding to the VEGF promoter region, also plays key roles on VEGF activity. Some scholars suggested that thalidomide inhibits the binding of the transcription factor SP1 to the promoter of the VEGF gene, which affects the production of VEGF and reduces the formation of blood vessels (Drucker et al., 2003). What is the relationship between STAT3 and SP1 on VEGF activity? It is worthy attention in future study.

## Data Availability Statement

The original contributions presented in the study are included in the article/Supplementary Material, further inquiries can be directed to the corresponding author.

## Ethics Statement

The animal study was reviewed and approved by the Ethics Committee of Shanghai General Hospital.

## Author Contributions

NZ and LW performed all the *in vitro* experiments and wrote the manuscript. JJ and LG performed the RT-PCR and western blot experiments. HGo, XW, MY, and HGa helped with the writing and revision of the manuscript. WY conceptualized and supervised the entire project. All authors contributed to the article and approved the submitted version.

## Conflict of Interest

The authors declare that the research was conducted in the absence of any commercial or financial relationships that could be construed as a potential conflict of interest.

## Publisher’s Note

All claims expressed in this article are solely those of the authors and do not necessarily represent those of their affiliated organizations, or those of the publisher, the editors and the reviewers. Any product that may be evaluated in this article, or claim that may be made by its manufacturer, is not guaranteed or endorsed by the publisher.
