# Supplementary material for: Thalidomide Suppresses Angiogenesis Through the Signal Transducer and Activator of Transcription 3/SP4 Signaling Pathway in the Peritoneal Membrane
Source: Front Physiol. 2021 Sep 3;12:712147. doi: 10.3389/fphys.2021.712147 (PMC8446434; doi:10.3389/fphys.2021.712147)
Supplement: Supplementary file 1 [file Table_1.DOCX]

Table 1 The qRT-PCR primer sequence information.

| Gene | Upstream primer（5’-3’） | Downstream primer（5’-3’） |
| --- | --- | --- |
| *Actin* | CATCACTGCCACCCAGAAGACTG | ATGCCAGTGAGCTTCCCGTTCAG |
| *VEGF* | AGGGCAGG ATCCACGTGC CCATT | CAAAAGATACATCTCATAAATAGT |
| *VEGFR2* | CCA TTATGACAAC ACAGCAGGAA | GTCTGGTTGTCATCTGGGATTAC |
| *VEGFR3* | GAGTTCTGC CAGCGGCTGA GAGA | CCAGGATCTCCACCAGCTCCGAG |
| *STAT3* | CA GAGGGTGCTT ACAACCTTGA | CCAGAATCAGAAGTATCCCAGC |
| *SP4* | AGGTTTATGCGGAGTGATCATCTC | CTCTGTAACAGATGAGTCCAGTT |
